# Case of *Plasmodium knowlesi* Malaria in Poland Linked to Travel in Southeast Asia

**DOI:** 10.3201/eid2509.190445

**Published:** 2019-09

**Authors:** Szymon P. Nowak, Paweł Zmora, Łukasz Pielok, Łukasz Kuszel, Ryszard Kierzek, Jerzy Stefaniak, Małgorzata Paul

**Affiliations:** Poznań University of Medical Sciences, Poznań, Poland (S.P. Nowak, Ł. Pielok, Ł. Kuszel, J. Stefaniak, M. Paul);; Institute of Bioorganic Chemistry, Polish Academy of Sciences, Poznań (P. Zmora, R. Kierzek)

**Keywords:** malaria, Plasmodium knowlesi, Sumatra, travel medicine, imported diseases, parasites, Poland, Southeast Asia, Indonesia, central Europe

## Abstract

We report a case of *Plasmodium knowlesi* malaria imported to central Europe from Southeast Asia. Laboratory suspicion of *P. knowlesi* infection was based on the presence of atypical developmental forms of the parasite in Giemsa-stained microscopic smears. We confirmed and documented the clinical diagnosis by molecular biology techniques.

The simian malaria parasite, *Plasmodium knowlesi*, is an emergent public health threat for persons traveling to Southeast Asia (*1*). We report a case of *P. knowlesi* malaria imported to central Europe from Southeast Asia.

On June 25, 2018, a 27-year-old woman returned to Poland after an 8-month tourist stay in Southeast Asia (Appendix Figure 1). The patient did not use malarial chemoprophylaxis during her travels. While in Sumatra, Indonesia, she experienced 2 episodes of subfebrile body temperature of <38°C. After returning to Poland, she reported having general malaise, weakness, chills, and a low-grade fever. She consulted a family physician, who diagnosed pharyngitis and recommended empiric antimicrobial drug therapy, cephalosporin combined with a fluoroquinolone, which provided no clinical improvement. After another episode of fever (temperature 39°C), she sought treatment at the regional hospital in Racibórz, Poland. Basic laboratory tests revealed leucopenia, thrombocytopenia, and elevated levels of C-reactive protein and procalcitonin. The patient did not have any chronic diseases or drug allergies. She was not pregnant, and her family history was unremarkable.

On July 5, 2018, the patient was transferred to the Department of Tropical and Parasitic Diseases, Poznań University of Medical Sciences, Poznań, Poland, because of high fever. At admission, on day 5 of her illness, she was conscious and responded logically. Her clinical status was stable. She was febrile (temperature 40°C) and experiencing hypotension (91/68 mm Hg), chills, headache, weakness, malaise, and tachycardia (110 bpm) but did not have signs of multiorgan failure. Laboratory analyses showed mild normocytic anemia (hemoglobin 10.3 g/dL, hematocrit 29.0%, and erythrocyte count 3.34 × 10^12^ cells/L); low levels of platelets (22 × 10^9^/L), leukocytes (2.13 × 10^3^/μL), neutrophils (0.76 × 10^3^/μL), and lymphocytes (1.01 × 10^3^/μL); marked elevation of inflammatory markers C-reactive protein (66.3 mg/L) and procalcitonin (0.67 ng/mL); a high concentration of D-dimers (6.48 × 10^3^ mg/mL); slightly prolonged prothrombin time (12.9 s); and elevated lactate dehydrogenase level (249 U/L).

Staff examining the first thick and thin blood films during screening in the emergency department reported an “atypical mixed infection with *P. vivax* and *P. malariae* with a strange morphology of the parasites” and a low parasitemia of 0.3%. A reference microscopic analysis performed at the Department of Tropical and Parasitic Diseases, Poznań University of Medical Sciences, showed infected erythrocytes of normal size and shape with a lack of Schuffner stippling and Maurer’s cleft. We observed multiple young trophozoites in the erythrocytes, with a delicate, thin ring of cytoplasm. Some also had narrow band shapes. In addition, we saw mature schizonts with <16 merozoites, large round gametocytes, and notable amounts of hemozoin pigment (Appendix Figure 2). ELISA revealed a high level of *Plasmodium* sp. IgM/IgG (52 U/mL), but we could not identify the *Plasmodium* species from these features. We later used PCR to confirm *P. knowlesi* infection from peripheral blood collected in EDTA tubes and frozen at –20°C. In brief, we extracted DNA from a 1.2-mL venous blood sample by using an automated nucleic acid extractor, MagCore HF16 Plus, with a MagCore genomic DNA large volume whole blood kit (RBC Bioscience Corp., https://www.rbcbioscience.com), according to standard protocol. To identify the *Plasmodium* species, we used nested PCR according to Komaki-Yasuda et al. (*2*). In patients with previously described *P. falciparum* malaria, we have observed a specific band for the parasite. We did not observe this band in the case-patient’s sample, suggesting infection with another *Plasmodium* species. The *P. vivax* primers did not yield amplification, but the *P. knowlesi* oligos resulted in clear bands, indicating that this patient was infected with *P. knowlesi* (Figure). In addition, the *P. knowlesi* band diminished after malarial therapy, demonstrating treatment efficacy.

**Figure:**
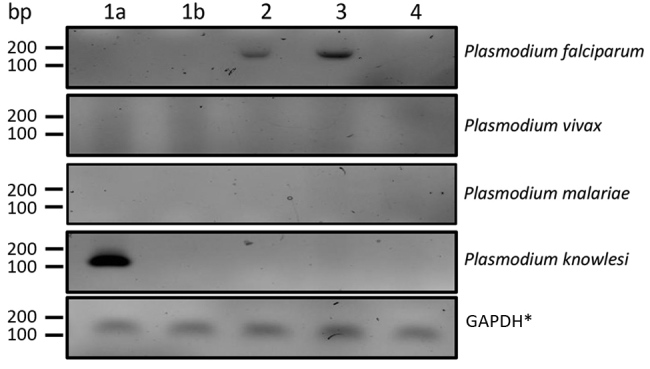
Nested PCR of *Plasmodium knowlesi* DNA isolated from a patient in Poland with recent travel to Southeast Asia. Lane 1a, patient sample from day of admission; lane 1b, patient sample taken 11 days after implementing malarial treatment; lanes 2 and 3, samples taken from patients previously diagnosed with *Plasmodium falciparum* malaria; lane 4, sample from an afebrile person from Poland with no history of travel to tropical countries. *GAPDH, glyceraldehyde 3-phosphate dehydrogenase.

 On the basis of the patient’s travel history, clinical signs and symptoms, test results, and World Health Organization guidelines (*3*), we diagnosed uncomplicated *P. knowlesi* infection. The patient received oral artemether and lumefantrine combined with intravenous doxycycline and the parasites cleared in microscopic smears within 4 days. The patient’s fever subsided, her blood morphology and biochemistry parameters improved, and her levels of inflammatory and coagulation system markers decreased. In addition, PCR was negative for *P. knowlesi* DNA in peripheral blood after treatment. During a 3-month follow-up period, morphological and biochemical laboratory parameters all normalized, and the level of *Plasmodium*-specific antibodies diminished to <28 U/mL.

In conclusion, we describe a rare case of *P. knowlesi* infection imported to Poland in a traveler returning from Southeast Asia. Previous research studies report imported cases of *P. knowlesi* malaria in travelers returning to other countries in western and northern Europe, including Spain, Italy, France, Germany, and Sweden (*4*–*10*). Travelers from Poland increasingly choose Southeast Asia as a common and popular destination. With that in mind, parasitology laboratories in Poland could diagnose *P. knowlesi* more often as an etiologic agent of tropical malaria. The ambiguous morphology and low number of parasites seen in microscopy make diagnosing *P. knowlesi* difficult. Proper diagnosis relies on thorough epidemiology, including travel history, augmented with molecular biology techniques.

Appendix Additional information on a case of *Plasmodium knowlesi* malaria in a patient in Poland with recent travel to Southeast Asia.
