# Supplementary material for: Case of Plasmodium knowlesi Malaria in Poland Linked to Travel in Southeast Asia
Source: Emerg Infect Dis. 2019 Sep;25(9):1772–3. doi: 10.3201/eid2509.190445 (PMC6711224; doi:10.3201/eid2509.190445)
Supplement: Appendix — Additional information on a case of Plasmodium knowlesi malaria in a patient in Poland with recent travel to Southeast Asia. [file 19-0445-Techapp-s1.pdf]

Article DOI: https://doi.org/10.3201/eid2509.190445

Case of *Plasmodium knowlesi* Malaria in Poland Linked to Travel in Southeast Asia

Appendix

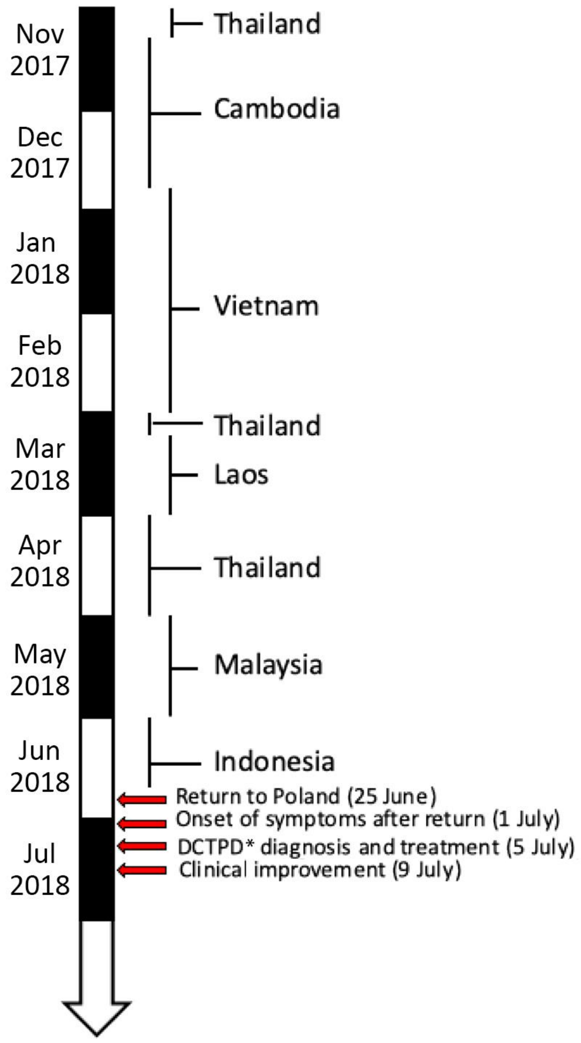

Appendix Figure 1. Timeline of case-patient's history of travel in Southeast Asia, onset of symptoms, and treatment for *Plasmodium knowlesi* malaria in Poland. *DCTPD, Department and Clinic of Tropical and Parasitic Diseases.

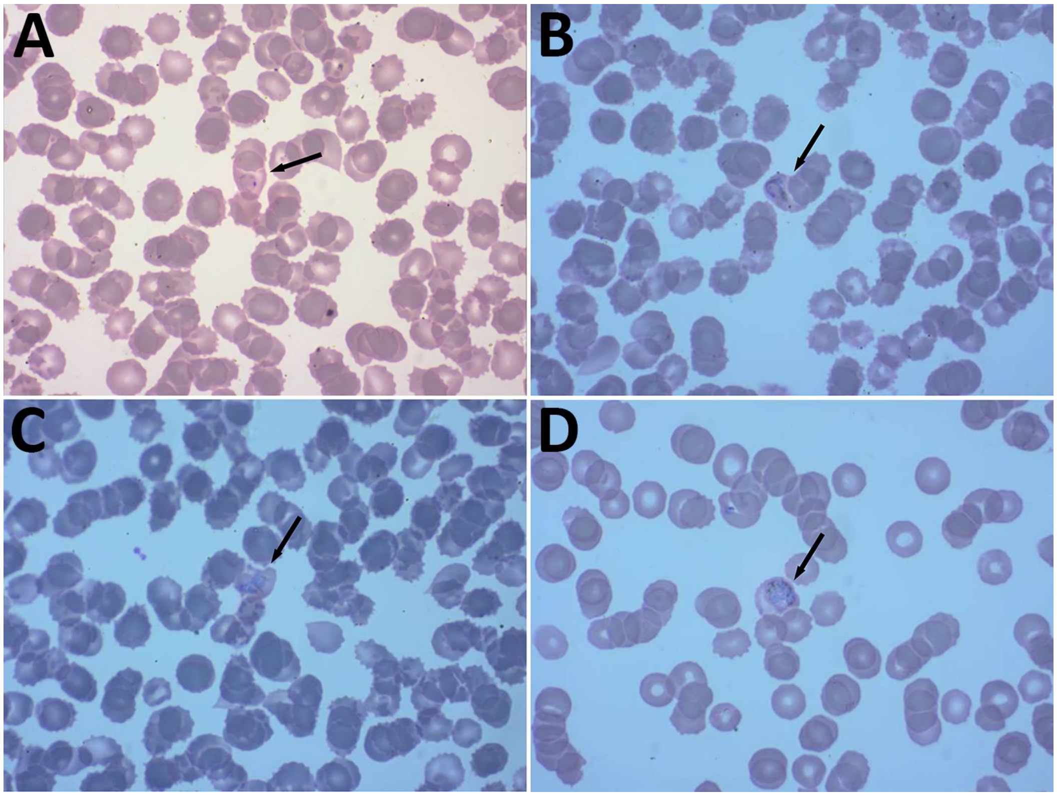

Appendix Figure 2. Microscopic morphology of *Plasmodium knowlesi* in Giemsa-stained thin blood films from a patient returning to Poland from travel in Southeast Asia. A) Arrow indicates early trophozoites with a delicate, thin ring of blue cytoplasm. B) Arrow indicates mature trophozoite. C) Arrow indicates immature schizont forming an equatorial band shape. D) Arrow indicates macrogametocyte with eccentric compact chromatin and scattered dark pigment of hemozoin in a blue cytoplasm.
